# Cell Death Induction and Protection by Activation of Ubiquitously Expressed Anion/Cation Channels. Part 2: Functional and Molecular Properties of ASOR/PAC Channels and Their Roles in Cell Volume Dysregulation and Acidotoxic Cell Death

**DOI:** 10.3389/fcell.2021.702317

**Published:** 2021-07-09

**Authors:** Yasunobu Okada, Kaori Sato-Numata, Ravshan Z. Sabirov, Tomohiro Numata

**Affiliations:** ^1^National Institute for Physiological Sciences (NIPS), Okazaki, Japan; ^2^Department of Physiology, School of Medicine, Aichi Medical University, Nagakute, Japan; ^3^Department of Physiology, Kyoto Prefectural University of Medicine, Kyoto, Japan; ^4^Department of Physiology, School of Medicine, Fukuoka University, Fukuoka, Japan; ^5^Japan Society for the Promotion of Science, Tokyo, Japan; ^6^Laboratory of Molecular Physiology, Institute of Biophysics and Biochemistry, National University of Uzbekistan, Tashkent, Uzbekistan

**Keywords:** cell volume regulation, necrotic volume increase, acidotoxic cell death, ischemia, anion channel, ASOR, PAC, hypothermia

## Abstract

For survival and functions of animal cells, cell volume regulation (CVR) is essential. Major hallmarks of necrotic and apoptotic cell death are persistent cell swelling and shrinkage, and thus they are termed the necrotic volume increase (NVI) and the apoptotic volume decrease (AVD), respectively. A number of ubiquitously expressed anion and cation channels play essential roles not only in CVR but also in cell death induction. This series of review articles address the question how cell death is induced or protected with using ubiquitously expressed ion channels such as swelling-activated anion channels, acid-activated anion channels, and several types of TRP cation channels including TRPM2 and TRPM7. In the Part 1, we described the roles of swelling-activated VSOR/VRAC anion channels. Here, the Part 2 focuses on the roles of the acid-sensitive outwardly rectifying (ASOR) anion channel, also called the proton-activated chloride (PAC) anion channel, which is activated by extracellular protons in a manner sharply dependent on ambient temperature. First, we summarize phenotypical properties, the molecular identity, and the three-dimensional structure of ASOR/PAC. Second, we highlight the unique roles of ASOR/PAC in CVR dysfunction and in the induction of or protection from acidotoxic cell death under acidosis and ischemic conditions.

## Introduction

Control of the cell volume in animal cells is essential for their survival. Even under acute anisotonic conditions, animal cells can shortly regulate their volume by coping with osmotic cell shrinkage by the regulatory volume increase (RVI) and with osmotic cell swelling by the regulatory volume decrease (RVD). RVI and RVD are attained by transmembrane water fluxes driven by gaining NaCl and losing KCl from extracellular and intracellular solutions, respectively (see Reviews: Lang et al., 1998; Okada, 2004; Hoffmann et al., 2009). Since these fundamental functions are conserved throughout evolution in animal cells irrespective of cell types for cell survival, the mechanisms of cell volume regulation (CVR) predominantly involve most ubiquitously expressed anion and cation channels. These ubiquitous volume-regulatory ion channels include volume-activated or volume-correlated anion channels (Okada et al., 2019), stretch-activated transient receptor potential (TRP) cation channels (Liu and Montell, 2015), and cell shrinkage-activated, hypertonicity-induced cation channel (HICC) (Wehner et al., 2003, 2006). These volume-regulatory ion channels also play protective roles against cell injury and death under osmotic stress.

Cell volume dysregulation leads to cell death. Actually, major hallmarks of necrotic and apoptotic cell death are persistent cell swelling and shrinkage, and thus they are called the necrotic volume increase (NVI) (Barros et al., 2001; Okada et al., 2001) and the apoptotic volume decrease (AVD) (Maeno et al., 2000), respectively. NVI and AVD are attained by water fluxes driven by the net gain of ambient NaCl and the net loss of cellular KCl, respectively. Thus, cell death is initiated by impairments or dysfunction of CVR mechanisms. Common pathological situations are injuries induced by ischemia or hypoxia and that followed by reperfusion or re-oxygenation, and they cause a variety of tissue stress including osmotic perturbation, production of reactive oxygen species (ROS), and acidic overload. ROS were shown to activate VSOR (Browe and Baumgarten, 2004; Shimizu et al., 2004; Varela et al., 2004) as well as TRPM7 and TRPM2 (Miller, 2006). It is noted that ROS were recently found not only to activate but also to inactivate VSOR activity in a manner dependent on the LRRC8 subunit composition (Gradogna et al., 2017). Acidity directly activates one type of ubiquitous anion channel which is called the acid-sensitive outwardly rectifying (ASOR) anion channels (Wang et al., 2007) or the proton-activated chloride (PAC) channel (Yang et al., 2019). Acidity is also known to rapidly augment activity of ubiquitously expressed TRPM7 cation channels (Jiang et al., 2005; Numata et al., 2019). Thus, altered activities of VSOR/VRAC and ASOR/PAC anion channels as well as of TRPM2 and TRPM7 cation channels are implicated in CVR dysfunction that eventually leads to cell death. In the previous Part 1 article (Okada et al., 2020), we summarized the roles of VSOR/VRAC activities in CVR and cell death induction. We here review the roles of ASOR/PAC activity in the present article (as Part 2), and will review the roles of TRPM2 and TRPM7 activities in the next article (as Part 3) in induction of CVR and cell death as well as protection from cell death.

## Phenotypical Properties and Molecular Identity of ASOR/PAC

### ASOR/PAC Activated Under Acidotic Conditions

Under physiological conditions, the pH of our body fluid is ordinarily maintained at 7.35–7.45, and the intracellular pH value is kept at 7.0–7.4 in animals, except for the following particular tissues. The surface of stomach exhibits highest acidity with the pH value of 1.35–3.5, and those of the vagina and skin are considerably acidic with their pH values of 4.0–5.0 and 4.0–6.5, respectively, in order to protect against microbial infection and overgrowth (García-Closas et al., 1999; Boelsma et al., 2003). In addition, osteoclasts secrete protons during bone resorption, thereby creating acidic microenvironments with pH of below 5.5 (Kato et al., 2013). Also, the pH value is only temporarily lowered in the vicinity of the exocytotic site of secretory granules which are very acidic with pH of 5.5 or less (Paroutis et al., 2004). It is plausible that some special protection mechanisms, such as the mucus-bicarbonate barrier-shield for the stomach surface, are installed at such physiologically acidic sites.

Under pathological conditions, acidity is often found in most tissues, especially at inflammatory sites due to proton production in filtrated neutrophils and macrophages and due to the presence of short-chain fatty acids that are by-products of bacterial metabolism (Jacobus et al., 1977; Lardner, 2001) and in tumor tissues due to the Warburg effects in cancer cells (Kallinowski and Vaupel, 1986; Martin and Jain, 1994; Kraus and Wolf, 1996; Kato et al., 2013). Also, acidity is induced in the tissues subjected to ischemia, because protons are produced when blood flow to a tissue is reduced or abolished, and then available ATP is hydrolyzed and the oxidized form of nicotinamide-adenine dinucleotide (NAD^+^) is reduced to NADH (by the reaction of RH_2_ + NAD^+^ → NADH + H^+^ + R) (Zeng and Xu, 2012). Especially, in the brain, extracellular pH may fall below 6 during ischemia, seizure, and hyperglycemic insults (Kraig et al., 1983; Mutch and Hansen, 1984; Rehncrona, 1985; Smith et al., 1986; Siesjö, 1988).

Extracellular acidification is known to directly activate several types of cation channels including TRPM7 expressed ubiquitously (Jiang et al., 2005; Numata et al., 2019) and the acid-sensing ion channel (ASIC) expressed predominantly in the central and peripheral nervous systems (Waldmann et al., 1997, 1999; Gründer and Chen, 2010). Under such acidic conditions, extracellular protons are also known to activate a specific type of anion channel, ASOR (Wang et al., 2007), also called PAC (Yang et al., 2019) or the proton-activated outwardly rectifying anion channel (PAORAC) (Lambert and Oberwinkler, 2005). Here, we call ASOR/PAC or simply ASOR. Since ASOR activity was first discovered in rat Sertoli cells in 2003 (Auzanneau et al., 2003), the functional expression of this channel has been found in a wide variety of cell types, as listed in Table 1, including epithelial cells and endothelial cells as well as blood, bone, muscle, neuronal and glial cells. Thus, it appears that ASOR is an acid-sensitive anion channel ubiquitously expressed in mammals.

**TABLE 1:** Cell types exhibiting functional expression of ASOR/PAC channel activity.

Cell Types	References
**Epithelial**	
Human cervical HeLa	Nobles et al., 2004; Wang et al., 2007; Sato-Numata et al., 2013, 2016, 2017; Ullrich et al., 2019; Yang et al., 2019
Human kidney HEK293	Nobles et al., 2004; Lambert and Oberwinkler, 2005; Kajiya et al., 2009; Matsuda et al., 2010a; Sato-Numata et al., 2013; Drews et al., 2014; Capurro et al., 2015; Ullrich et al., 2019; Yang et al., 2019
Human bronchial HBE	Nobles et al., 2004; Capurro et al., 2015
Human colonic Caco-2	Nobles et al., 2004
Human tracheal HHE	Nobles et al., 2004
Human pancreatic CFPAC	Capurro et al., 2015
Human nasopharyngeal	Wang et al., 2012
Mouse kidney MDCT, mIMCD-3	Valinsky et al., 2017
Mouse mammary gland C127	Okada et al., 2017
Rat thyroid FRT	Capurro et al., 2015
Rat insulinoma INS-1E	Osei-Owusu et al., 2020
**Endothelial**	
Human umbilical vein	Ma et al., 2008
**Blood**	
Human erythrocytes	Kucherenko et al., 2009
Human monocytes	Shi et al., 2009
Human monocytic THP-1	Fu et al., 2013
Mouse RAW264.7	Shi et al., 2009; Ohgi et al., 2011; Capurro et al., 2015
**Bone**	
Mouse osteoclasts	Ohgi et al., 2011
Human chondrocyte OUMS-27	Kurita et al., 2015
Human chondrocyte C28/D2	Kittl et al., 2020
Human primary chondrocytes	Kittl et al., 2020
**Muscle**	
Mouse and guinea pig cardiac	Yamamoto and Ehara, 2006
Mouse vascular smooth muscle	Matsuda et al., 2010a
**Neuronal**	
Rat pheochromocytoma PC-12	Nobles et al., 2004
Human neuroblastoma SK-N-MC	Capurro et al., 2015
Mouse cortical neurons	Sato-Numata et al., 2014
Rat cortical neurons	Osei-Owusu et al., 2020
**Glial**	
Mouse astrocytes	Lambert and Oberwinkler, 2005
Mouse microglial BV-2	Kittl et al., 2019

### Phenotypical Properties of ASOR/PAC Currents

Based on nine systematic ASOR studies published between 2003 and 2019 (Auzanneau et al., 2003; Nobles et al., 2004; Lambert and Oberwinkler, 2005; Yamamoto and Ehara, 2006; Wang et al., 2007; Ma et al., 2008; Fu et al., 2013; Capurro et al., 2015; Kittl et al., 2019), ASOR currents are phenotypically characterized by rapid and reversible activation upon severe extracellular acidification, strong outward rectification, activation time course during application of positive voltage pulses, anion selectivity, and marked sensitivity to 4,4′-diisothiocyanatostilbene-2,2′-disulfonic acid (DIDS). It appears that ASOR activation is induced by protons from the extracellular side, because extracellular acidification readily brought about activation of ASOR currents even in the intracellular presence of a high concentration pH buffer (100 mM HEPES) (Lambert and Oberwinkler, 2005), and because intracellular acidification (down to pH 4.5) never induced ASOR activation (Kajiya et al., 2009; Kittl et al., 2019) and never affected ASOR current activation induced by extracellular acidification (Kittl et al., 2019).

The extracellular pH value for half-maximum activation (EC_50_) somewhat varies depending on the cell types and was found to increase from 4.6, 4.9, and 5.0 to 5.2, 5.4, and 5.5 by raising temperature from room temperature (at ∼25°C) to 35–37°C in HeLa cells (Sato-Numata et al., 2013), cortical neurons (Sato-Numata et al., 2014), and HEK293 cells (Yang et al., 2019), respectively. The ASOR current amplitude also increases with increasing temperature, and the 10° temperature coefficient (Q_10_) value, which is an indicator of temperature sensitivity and to be evaluated from the slope of a linear part of the Arrhenius plot, was found to sharply increase from 1.2 to 8.8 in HeLa cells (Sato-Numata et al., 2013) and from 2.2 to 5.6 in cortical neurons (Sato-Numata et al., 2014), when temperature was raised from room temperature to body-like warm temperature.

The halide anion permeability sequence of ASOR channels was found to be I^–^ > Br^–^ > Cl^–^ (Eisenman’s sequence I) in many cell lines (Nobles et al., 2004; Lambert and Oberwinkler, 2005; Wang et al., 2007; Capurro et al., 2015), whereas Br^–^ > I^–^ > Cl^–^ (Eisenman’s II) was in cardiac myocytes (Yamamoto and Ehara, 2006) and Cl^–^ > Br^–^ > I^–^ (Eisenman’s IV) was in Sertoli (Auzanneau et al., 2003) and monocytic THP-1 (Fu et al., 2013) cells.

The single-channel conductance was so far recorded by only two groups and shown to be less than 10 pS at physiological membrane potentials with two different types of voltage dependence. Lambert and Oberwinkler (Lambert and Oberwinkler, 2005) observed its increases from 4 to 7 pS at negative potentials and around 10 pS at +46 mV, respectively, in HEK293 cells and thereby explained outward rectification of whole-cell currents mainly by voltage-dependent changes in the unitary conductance. On the other hand, Wang et al. (2007) showed that it is 4.8 pS in the range of voltage between −80 and +80 mV in HeLa cells, and that the mean number of open channels (*NP*_o_) of these unitary currents increases with increasing the voltage. They thereby explained outward rectification of macroscopic currents mainly by voltage-dependent changes of the *NP*_o_ value.

There has been an accumulating body of pharmacological information about ASOR blockers, although they are more or less non-specific. DIDS is a most well-established ASOR blocker which is effective at submicromolar to micromolar concentrations (Nobles et al., 2004; Lambert and Oberwinkler, 2005; Yamamoto and Ehara, 2006; Wang et al., 2007; Ma et al., 2008; Shi et al., 2009; Fu et al., 2013; Sato-Numata et al., 2014; Capurro et al., 2015; Valinsky et al., 2017; Kittl et al., 2019) with exhibiting the IC_50_ values of 2.9 μM (Lambert and Oberwinkler, 2005) or 0.5 μM (Capurro et al., 2015) in HEK293 cells, 0.12 μM in HeLa cells (Wang et al., 2007), and 4.7 μM in cortical neurons (Sato-Numata et al., 2014). ASOR activity was shown to be also sensitive to phloretin (Wang et al., 2007; Sato-Numata et al., 2014) with IC_50_ of 17.5 μM, to niflumic acid (Nobles et al., 2004; Yamamoto and Ehara, 2006; Capurro et al., 2015; Sato-Numata et al., 2016) with IC_50_ of 11.0 μM (Sato-Numata et al., 2016), to flufenamic acid at 100 μM (Lambert and Oberwinkler, 2005; Valinsky et al., 2017), and to diphenylamine-2-carboxylate (DPC) at 200–500 μM (Nobles et al., 2004; Capurro et al., 2015). ASOR currents were reported to be inhibited by many other chemicals including suramin, arachidonic acid, and *R*-(+)-[(2-*n*-butyl-6,7-dichloro-2-cyclopentyl-2,3-dihydro-1-oxo-1*H*-inden-5-yl)oxy] acetic acid (DIOA) with IC_50_ of 0.05, 8.9, and 1.9 μM, respectively (Sato-Numata et al., 2016) as well as by an HMG-CoA reductase inhibitor, simvastatin, with IC_50_ of 13.8 μM (Shi et al., 2009) and also by steroidal pregnenolone sulfate (PS) and dehydroepiandrosterone (DHEA) sulfate with IC_50_ of 5.1 and 25.7 μM, respectively (Drews et al., 2014). Glibenclamide was reported to be an effective ASOR blocker in cardiac myocytes at 100 μM (Yamamoto and Ehara, 2006) but be little effective to block ASOR currents in HEK293 cells (Capurro et al., 2015). Also, sensitivity of ASOR currents to 5-nitro-2-(3-phenylpropylamino)-benzoic acid (NPPB) was observed at 100 μM in HEK293 cells (Capurro et al., 2015) and in kidney epithelial cells (Valinsky et al., 2017) but never in HeLa cells (Sato-Numata et al., 2016). A relatively VSOR-selective blocker, 4-(2-butyl-6,7-dichloro-2-cyclopentylindan-1-on-5-yl)oxybutyric acid (DCPIB), was shown to be totally ineffective at blocking ASOR currents in HeLa cells (Sato-Numata et al., 2016) and microglial BV-2 cells (Kittl et al., 2019) at 10 μM but was found to be able to suppress not only VSOR but also ASOR currents in immortalized human chondrocyte-derived C28/I2 cells at 10 μM (Kittl et al., 2020).

There has been only fragmental information about intracellular regulators for ASOR activation. ASOR activity was found to be independent of intracellular Ca^2+^ (Auzanneau et al., 2003; Nobles et al., 2004; Yamamoto and Ehara, 2006; Kittl et al., 2019), Mg^2+^ (Lambert and Oberwinkler, 2005), and ATP (Yamamoto and Ehara, 2006; Capurro et al., 2015). ASOR activation was not affected by a non-hydrolyzable GDP analog, guanosine-5′-O-(2-thiodiphosphate) (GDPβS) (Yamamoto and Ehara, 2006), and a broad-spectrum protein kinase inhibitor, 2-methylpiperazine dihydrochloride (H-7) (Capurro et al., 2015), excluding involvements of G-protein-coupled receptor (GPCR) and protein kinases in ASOR activation. Pharmacological studies with a ROS scavenger (Fu et al., 2013), genistein (Capurro et al., 2015), and wortmannin (Capurro et al., 2015) suggested involvements of ROS, protein tyrosine phosphatase (PTP), and phosphoinositide 3-kinase (PI3K) in the regulation of ASOR activity.

Collectively, ASOR is the temperature-dependent anion-selective channel ubiquitously expressed and activated by extracellular strong acidity. ASOR currents exhibit phenotypical biophysical properties with a small single-channel conductance, sharp outward rectification, and activation kinetics in response to positive step pulses, as well as pharmacological properties such as marked sensitivity to DIDS, as summarized in Table 2.

**TABLE 2:** Comparison of properties of ASOR/PAC and VSOR/VRAC channel activities.

	ASOR/PAC	VSOR/VRAC
**Pore properties**		
Unitary conductance	Small (4–10 pS)	Intermediate (10–90 pS)
Outward rectification	Sharp	Mild
Gating at positive voltages	Activation kinetics	Inactivation kinetics
**Activation factor**		
Strong acidity	Activated	Suppressed
Cell swelling	Insensitive	Activated
**Regulatory factor**		
Cytosolic ATP	Independent	dependent
Cytosolic Mg^2+^	Insensitive	Sensitive
**Pharmacology**		
DIDS sensitivity	Stronger	Milder
DCPIB sensitivity	None/little*	Strong
Extracellular ATP sensitivity	None	Voltage-dependently blocked

*Except for human chondrocyte C28/I2 cells (see in the text).

### Molecular Identity of the ASOR/PAC Core Component

Until very recently, the molecular identity of ASOR channel was not determined. ASOR and VSOR channels were initially hypothesized to share the identical molecular entity and rapidly switch between two different manifestations depending on the extracellular pH level (Nobles et al., 2004). Both channel currents actually exhibit similar peak amplitudes, outward rectification, and sensitivity to DIDS. However, they show essentially different biophysical properties, as summarized in Table 2, such as one-digit smaller unitary conductance, much steeper outward rectification, and activation (not inactivation) kinetics, compared to known properties of VSOR currents as summarized in the preceding Part 1 article (Okada et al., 2020). In addition, between ASOR and VSOR currents, there are distinct differences in physiological and pharmacological properties (Table 2), such as independence of intracellular ATP (Yamamoto and Ehara, 2006; Capurro et al., 2015), and insensitivity to cytosolic high concentration Mg^2+^ (Lambert and Oberwinkler, 2005), over one-order higher sensitivity to DIDS, little sensitivity to a VSOR-specific blocker DCPIB, and insensitivity to extracellular ATP (Sato-Numata et al., 2016; Kittl et al., 2019). Also, ASOR exhibits much higher sensitivity to suramin, DIOA, arachidonic acid and niflumic acid compared to VSOR (Sato-Numata et al., 2016). Moreover, it must be noted that ASOR and VSOR currents can be simultaneously activated and then develop in an additive fashion in an acidic and hypoosmotic solution (Lambert and Oberwinkler, 2005; Yamamoto and Ehara, 2006; Matsuda et al., 2010b; Kittl et al., 2020), strongly suggesting independent activation of ASOR and VSOR channel molecules. Recently, the leucine-rich repeats containing 8A (LRRC8A) was identified as the core component of VSOR (Qiu et al., 2014; Voss et al., 2014), and functional VSOR activity was shown to require, in addition to LRRC8A, at least one of LRRC8A paralogs (LRRC8B/C/D/E) (Voss et al., 2014; Syeda et al., 2016). Therefore, we examined a possible relationship between LRRC8 members and ASOR-related molecules by observing the effect of gene-silencing of each LRRC8 member on ASOR currents in HeLa cells. The ASOR currents were not affected by such single siRNA-mediated knockdown not only of LRRC8A (Sato-Numata et al., 2016; Okada et al., 2017) but also of LRRC8B/C/D/E (Sato-Numata et al., 2016). Moreover, the ASOR currents were affected neither by double knockdown of LRRC8C or 8D *plus* LRRC8E nor by triple knockdown of LRRC8C, 8D *plus* 8E and by quadruple knockdown of LRRC8B, 8C, 8D *plus* 8E (Sato-Numata et al., 2017). Thus, it appears that the ASOR molecule is distinct from the VSOR core molecule LRRC8A and its paralogs.

The second candidate proposed as the ASOR molecule was the ClC family member expressed in a wide range of organisms. Among their nine members expressed in mammals, ClC-3 to -7 are known to be mainly expressed in the intracellular endosomal-lysosomal system and function as Cl^–^/H^+^ antiporters, whereas ClC-1, -2, -Ka, and -Kb exist at the plasma membrane and function as Cl^–^ channels (Jentsch and Pusch, 2018). Although there is a lot of evidence against ClC-3 being the ASOR molecule, ClC-3 was previously proposed to serve as an ASOR channel at acidic pH (Matsuda et al., 2010a), based on the following observations: First, ASOR activity was downregulated in aortic vascular smooth muscle cells isolated from ClC-3 knockout mice. Second, ASOR currents recorded in HEK293 cells exposed to pH 4 solution were enhanced by ClC-3 overexpression in a manner sensitive to siRNA-mediated knockdown of ClC-3. Third, when the charge-neutralized E224A mutant of ClC-3 was overexpressed, ASOR currents became activated at neutral pH (=7.35). Fourth, a thiol reagent (MTSES) abolished ASOR currents in wild-type HEK293 cells but not in HEK293 cells overexpressing a ClC-3 mutant which lacks four extracellular cysteine residues. Subsequently, the “ASOR = ClC-3” hypothesis was supported by two studies in which suppressive effects of ClC-3 knockdown on the Cl^–^ currents activated by mild acidity were observed in human nasopharyngeal CNE-2Z cells (Wang et al., 2012) and in rat pancreatic acinar AR42J cells (Yang et al., 2020). However, the properties of these anionic currents look distinct from ASOR currents but rather similar to VSOR currents with exhibiting moderate, but not sharp, outward rectification and sensitivity not only to DIDS but also to NPPB (Wang et al., 2012; Yang et al., 2020) as well as inactivation, but not activation, kinetics at +80 mV and sensitivity to hypertonic cell shrinkage (Wang et al., 2012) and to a ROS scavenger (Yang et al., 2020). Also, these anionic currents were activated only by mild acidity (pH 7.1–6.6) but rather inhibited by strong acidity (pH 5.8) (Wang et al., 2012; Yang et al., 2020). It is noted that VSOR currents are known to exhibit such pH dependence as enhancement by weak acid (Arreola et al., 1995) and suppression by strong acid (Kittl et al., 2019, 2020; Table 2). Furthermore, it must be pointed out that these whole-cell recordings were performed by applying intracellular (pipette) and extracellular (bath) solutions with the same osmolarity, thus resulting in hypotonic stress caused by intracellular colloid osmotic pressure due to the endogenous presence of immobile macromolecular osmolytes within the cytosol. Contrary to this “ASOR = ClC-3” hypothesis, we showed that ASOR activity in HeLa cells was affected neither by siRNA-mediated molecular downregulation of ClC-3 nor by molecular overexpression of ClC-3 (Sato-Numata et al., 2013). Similarly, gene silencing of ClC-3 failed to affect ASOR currents in HEK293 cells (Capurro et al., 2015). Furthermore, molecular expression levels of ClC-3 mRNA and protein were found to be indistinguishable between ASOR activity-rich KB cells and its cisplatin-resistant subline KCP-4 cells largely lacking ASOR activity (Sato-Numata et al., 2013).

ClC-3 is predicted to have six isoforms, ClC-3a to -3f, with distinct *N*- and *C*-terminal amino acid sequences. The ClC-3 isoform, which was previously proposed to serve as ASOR (Matsuda et al., 2010a) and VSOR (Duan et al., 1997, 1999), corresponds to ClC-3a, the *N*-terminal short form of ClC-3. However, we showed that ClC-3a overexpressed in HEK293 cells exhibits Cd^2+^-sensitive, phloretin-insensitive anionic currents and the properties of which are distinct from those of ASOR and VSOR currents (Okada et al., 2014). ClC-3b, the *N*-terminal long form, was reported to function as a Cl^–^ channel activated by Ca^2+^/calmodulin-dependent protein kinase IIα when stably transfected in tsA cells (Huang et al., 2001). ClC-3e, the *C*-terminal variant of ClC-3b, was shown to serve as a CFTR-regulated outwardly rectifying anion channel when co-transfected with EBP50 in C127 cells (Ogura et al., 2002). ClC-3d was found to be a *C*-terminal variant of the short form with an *N*-terminal sequence identical to ClC-3a and mediate Cd^2+^-sensitive, phloretin-insensitive outwardly rectifying Cl^–^ currents, the properties of which are again distinct from those of ASOR and VSOR currents (Okada et al., 2014). It must be noted that ASOR currents were never suppressed by Cd^2+^ (Okada et al., 2014). Thus, it appears that ClC-3a and -3d are both distinct from the molecular entity of ASOR channel.

Another ClC member proposed to be a candidate for ASOR channel was ClC-7 (Diewald et al., 2002; Kajiya et al., 2009; Ohgi et al., 2011; Kurita et al., 2015), which is largely expressed in osteoclasts and functioning as an electrogenic Cl^–^/H^+^ antiporter exchanging Cl^–^ and H^+^ with a stoichiometry of 2:1 in their lysosomes and ruffled borders (Jentsch and Pusch, 2018). Diewald et al. (2002) first reported that acidic pH-activated Cl^–^ currents were augmented when rClC-7-cRNA was injected into *Xenopus laevis* oocytes and exhibited strong outward rectification and DIDS sensitivity. However, similar currents were, though much smaller, also observed in the mock-transfected oocytes in which ClC-7 protein was not detected (Diewald et al., 2002). Also, it is noted that the cRNA of Ostm1, which is a β-subunit required for ClC-7 activity (Leisle et al., 2011), was not co-injected in this study (Diewald et al., 2002). Then, such ASOR currents were observed in mouse osteoclasts and in ClC-7-overexpressing HEK293 cells, and the ASOR currents were found to be suppressed when transfected with ClC-7 mutants associated with autosomal dominant osteopetrosis type II (Kajiya et al., 2009). However, it must be stressed that HEK293 cells endogenously express ASOR activity, as noted above. This “ASOR = ClC-7” hypothesis was supported by the observations that three different polyclonal antibodies against ClC-7 suppressed ASOR currents recorded in mouse osteoclasts and Raw264.7 cells in which ClC-7 was stably overexpressed (Ohgi et al., 2011). However, it must be pointed out that one of antibodies against the epitope with R286 was found to work from the extracellular side in this study, although this part of ClC-7 is known to exist on the intracellular side (Zhang et al., 2020). On the other hand, supporting this “ASOR = ClC-7” hypothesis, endogenous ASOR activity was found to be suppressed by treatment with ClC-7 siRNA in human chondrocyte OUMS-27 cells (Kurita et al., 2015). However, at variance with this hypothesis, ClC-7 was shown to reside in the intracellular compartment but not at the plasma membrane in Sertoli cells (Auzanneau et al., 2003), and siRNA-mediated knockdown of ClC-7 failed to suppress endogenous ASOR currents in HEK293 cells (Capurro et al., 2015). Taken together, it is highly likely that ClC-7 is not the ASOR channel molecule by itself but may serve, if massively expressed, as an augmenting regulator of ASOR activity, presumably by providing protons via operation of Cl^–^/H^+^ antiports to nearby ASOR channel molecules.

TMEM16, also known as anoctamin (ANO), forms a family of 10 members (TMEM16A-H, J, K or ANO1-10). TMEM16A (ANO1) was recently demonstrated to represent the Ca^2+^-activated Cl^–^ channel (CaCC) independently by three groups (Caputo et al., 2008; Schroeder et al., 2008; Yang et al., 2008). Soon after this, TMEM16B (ANO2) was also shown to serve as the CaCC function (Pifferi et al., 2009; Stephan et al., 2009). TMEM16F (ANO6) was found to be a multifunctional protein serving not only as CaCC (Grubb et al., 2013; Shimizu et al., 2013) but also as a Ca^2+^-dependent phospholipid scramblase (Suzuki et al., 2010). The functions of other members are poorly understood, though TMEM16D-H, J, and K (ANO4-10) were shown to produce transient CaCC currents when overexpressed in HEK293 cells (Tian et al., 2012). Thus, there remained a possibility that some member may contribute to the molecular entity of ASOR. However, ASOR activity in HEK293 and HBE cells, which lack expression of TMEM16E and J, was found to be unaffected by gene silencing of TMEM16A, D, F, H, and K (Capurro et al., 2015), thereby ruling out their contributions to the ASOR channel formation.

In 2019, by an unbiased genome-wide RNA interference screening, the ASOR/PAC molecule was eventually identified to be TMEM206, which is a membrane-spanning protein displaying two transmembrane domains (TMDs), for the first time by Qiu’s group (Yang et al., 2019) and immediately thereafter by Jentsch’s group (Ullrich et al., 2019). Although TMEM206 has no sequence similarity to other ion channels, both groups concluded that TMEM206 provides the core component of ASOR, because CRISPR/Cas9-mediated gene deletion of TMEM206 abolished ASOR currents in HEK293 cells, and overexpression of TMEM206 in the TMEM206-knockout cells rescued the ASOR activity (Ullrich et al., 2019; Yang et al., 2019). Also, TMEM206 overexpression in wild-type HEK293 cells was found to augment their ASOR activity (Ullrich et al., 2019). Furthermore, primary cortical neurons isolated from TMEM206-knockout mice were shown to lack ASOR activity (Yang et al., 2019). It appears that TMEM206 forms the ion-conducting pathway or pore of ASOR, because the I307A mutant exhibited a reduction of I^–^ permeability compared to the wild-type TMEM206 (Yang et al., 2019), because replacement of non-charged L315 by negatively charged aspartate (L315D) resulted in the loss of anion selectivity (Ullrich et al., 2019), and more strikingly because a charge-reversing mutation, K319E, converted this activity from an anion-selective channel with P_Cl_/P_Na_ of 6.1 to a cation-selective channel with P_Cl_/P_Na_ of 0.05 (Ruan et al., 2020). In complete agreement with this conclusion, recently gene silencing of TMEM206 was shown to largely eliminate ASOR currents in rat insulinoma INS-1E cells and rat cortical neurons (Osei-Owusu et al., 2020). Molecular expression of TMEM206 was found in a wide variety of human tissues (Yang et al., 2019), being consistent with ubiquitous expression of functional ASOR activities (Table 1). Most recently, TMEM206 was found to represent a Cl^–^ conducting pathway in endosomes and regulate endosome pH, thereby controlling the trafficking and recycling of membrane receptors (Osei-Owusu et al., 2021). Thus, it appears that not only the plasma membrane but also the endosomal membrane exhibit expression of functional ASOR activities, although it is not known whether the endosomal ASOR/PAC activity is involved in induction of and/or protection from cell death.

### Three-Dimensional Structure of ASOR/PAC Core Component

**FIGURE 1:**
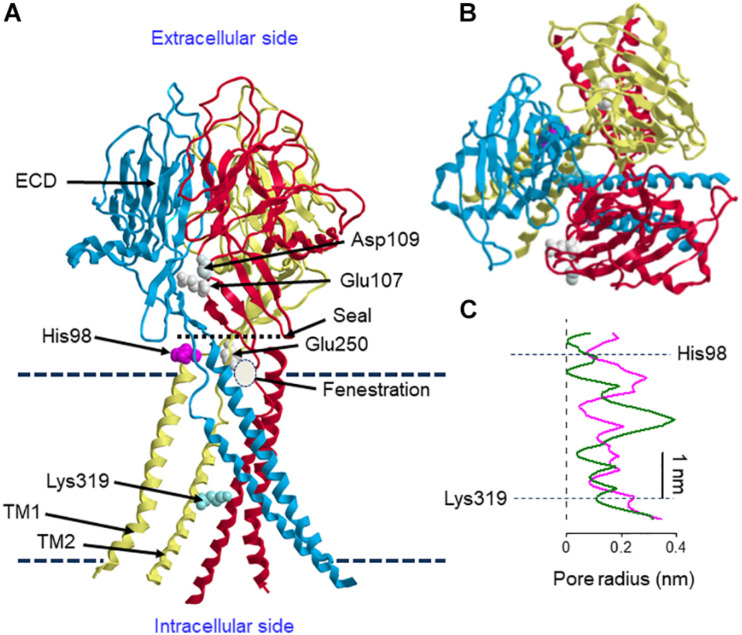
Structural features of human ASOR/PAC. **(A)** Side view of the structure of ASOR/PAC formed by trimeric TMEM206 at pH 8. Proton sensor amino acid, His98, and the selectivity filter determinant, Lys319, are shown in one of the subunits. Upon protonation, His98 is expected to move to the acidic pocket formed by Glu250 of the cognate subunit and Glu107 and Asp109 of the adjacent subunit. Upper and lower broken lines indicate extracellular and intracellular membrane-liquid boundaries, respectively. Positions of the seal, fenestration and membrane boundaries are approximate. **(B)** Its top view. **(C)** Pore radius at pH 8 (green) and pH 4 (magenta). The structure is drawn according to Ruan et al. (2020) using the 7jna.pdb file downloaded from https://www.rcsb.org/structure/7jna. The curves in panel **(C)** are produced by mimicking the curves of the Figure 3G of Ruan et al. (2020), modified with indicating the sites of His98 and Lys319, and scaled to roughly correspond to the channel structure in panel **(A)**.

The three-dimensional structure of the human ASOR/PAC channel has recently been established using cryo-electron microscopy (cryo-EM) of the recombinant TMEM206 channel protein reconstituted into the lipid nanodiscs and single-particle image analysis (Ruan et al., 2020). Figure 1 illustrates structural features of human ASOR/PAC channel at pH 8 according to Ruan et al. (2020). TMEM206 has two TMDs and shares membrane topology with ASIC (acid-sensitive Na^+^ channel) and ENAC (epithelial Na^+^ channel) (Ullrich et al., 2019; Yang et al., 2019). Likewise, the human ASOR/PAC channel exhibited a homo-trimeric structure (Figures 1A,B). An essentially similar cryo-EM structure of the pufferfish TMEM206 observed at pH 8 has recently been reported (Deng et al., 2021). The resting closed state at pH 8 displayed a ball-shaped extracellular domain (ECD) composed of fourteen β-strands and connected to a slim TMD consisted of two helices, TM1 and TM2 (Figure 1A). His98 located at the TM1-β1 linker provides a pH sensor for the ASOR channel. Comparison of structures at pH 8 and pH 4 showed that the protonated His98 residue at the ECD-TMD interface moves to the “acidic pocket” composed of Glu250 of the cognate subunit and Glu107 and Asp109 of the adjacent subunit at ECD. Thus, acid-induced movement of His98 becomes decoupled from the resting position interacting with the TM1-β1 linker and causes a large-scale rearrangement of TMD where TM1 exhibits a rotational movement with an angle to 64° and then switches its interacting partner from the cognate TM2 to the TM2 of an adjacent subunit. The ion-conducting pore is formed by the TM2 helices which contain both hydrophobic and hydrophilic residues including K319, and the protonated channel (at pH 4) has a wider hydrophilic pathway along the central pore axis in contrast to the narrower and less solvent-accessible pore of the deprotonated channel at pH 8 (Figure 1C). The deprotonated channel pore is occluded at several points. Although these obstacles are removed upon protonation, even the enlarged pore radius of 0.082 nm at the intracellular part of the pore does not allow the passage of chloride ions. Therefore, the cryo-EM-revealed structures at pH 8 and pH 4 may represent closed and pre-open or desensitizing states, respectively, and are both non-conductive. Interestingly, the channel has a “seal” at the ECD-TMD interface near the extracellular vestibule. Thus, the permeating ions cannot go through this vestibule. Instead, they may get into the pore lumen via “fenestrations,” small lateral openings at each subunit below the “seal” (Figure 1A). At pH 8, the fenestrations are small and lined with negatively charged residues, whereas at pH 4 they are wider and lined with positively charged amino acids and favor entering of anions. Similar lateral openings were also detected in the structures of ASIC (Gonzales et al., 2009) and P2X3 channels (Mansoor et al., 2016). Anionic selectivity and strong outward rectification are determined by the positively charged Lys319, since the charge-reversed mutant K319E was found to form a cation-selective channel and display inward rectification (Ruan et al., 2020). Similarly, the corresponding basic residue, Lys320, in the pufferfish TMEM206 has been recently shown to constitute the anion selectivity filter of pufferfish ASOR/PAC (Deng et al., 2021). The activation mechanism is not completely clear at present. Although His98 is a major determinant of the structural rearrangement upon protonation, its replacement with Arg or Ala did not abolish proton activation. Most profound effects were observed by the charge-reversed E107R mutation suggesting that electrostatic spatial arrangement of the acidic pocket is also important for the activation mechanism. Collectively, a recent cryo-EM structure study performed at pH 8 and pH 4 (Ruan et al., 2020) demonstrated that acid induces a marked conformational change in the interface between the TMD and the ECD as well as a rotational movement of the first TMD. However, it must be noted that the structure of the full open-state of ASOR/PAC channel still remains unexplored.

## Roles of ASOR/PAC in Induction of Cell Volume Changes and Cell Death Under Acidic and Ischemic Conditions

### Roles of ASOR/PAC in Acidic Cell Swelling

**FIGURE 2:**
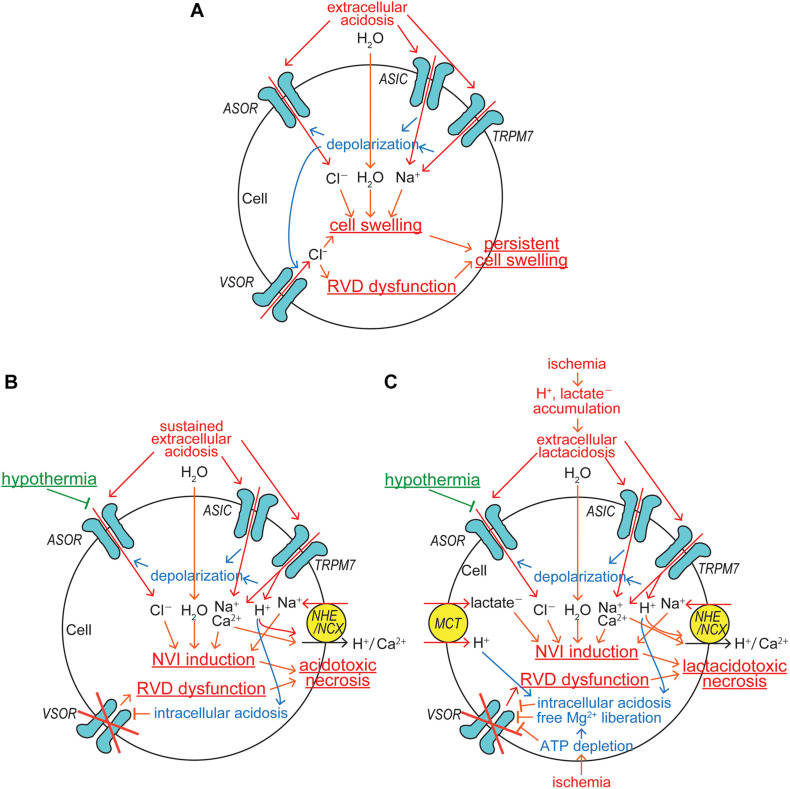
ASOR/PAC involvements in induction/protection of cell swelling and death under acidosis and ischemic conditions. **(A)** The mechanisms for induction of persistent cell swelling under acidosis. **(B)** The mechanisms for induction of acidotoxic necrosis and its hypothermic protection under sustained acidosis. **(C)** The mechanisms for induction of lactacidotoxic necrosis and its hypothermic protection under ischemic conditions (See the text for details).

Under acidosis, cells exhibit swelling. In light of following observations, it appears that acid-induced activation of ASOR anion channels, but not basal Cl^–^ conductance, is causatively involved in cell swelling induced by acidosis. First, acid-induced cell swelling was eliminated by application of ASOR blocking drugs in human epithelial HeLa cells (Wang et al., 2007; Ullrich et al., 2019). Second, neuronal cell swelling in response to acidosis was largely inhibited by ASOR blockers in mouse cortical neurons (Sato-Numata et al., 2014). Third, knockout of TMEM206 eliminated swelling responses to acidosis in human epithelial HEK293 cells (Ullrich et al., 2019). After reaching the peak swelling within several minutes, the cell volume tended to be slightly decreasing. Direct measurements of cell volume by Coulter counter (Wang et al., 2007) or of cell size by the cross-sectional area (CSA) evaluation (Sato-Numata et al., 2014) showed that after peak swelling, cells still exhibited sustained swelling under acidotic conditions, but never became shrunken below the normal cell volume level. In contrast, indirect measurements of cell volume by changes in the fluorescence of calcein loaded in the cells suggested that after peak swelling the cells became shrunken gradually in an undershooting manner (Ullrich et al., 2019). However, it must be pointed out that apparently undershooting cell shrinkage such indirectly observed cannot be explained only by parallel activation of ASOR and some acid-activated cation channel(s), because depolarization induced by activation of non-selective cation channels inevitably drives Cl^–^ influx via ASOR leading to cell swelling but not shrinkage. Thus, there remains a possibility that such apparent undershooting shrinkage was due to some side effect or quenching error of fluorescence measurements. To produce net increases in the cell volume during ASOR activation, parallel activation of the Na^+^-permeable cation channel is required. It is known that acid induces activation of two types of non-selective cation channels, ubiquitous TRPM7 (Jiang et al., 2005) and neuronal ASICs (Xiong et al., 2004; Yermolaieva et al., 2004). Activation of these non-selective cation channels induces not only Na^+^ influx but also cell depolarization, which gives rise to a driving force for ASOR-mediated Cl^–^ influx, thereby causing cell swelling (Figure 2A). Since ASIC is known to be rapidly deactivated during acid exposure (Gründer and Pusch, 2015), in nervous systems, this may account for a gradual diminishment of cell swelling after reaching the peak swelling. Acidic cell swelling thus elicited may activate VSOR anion channels. However, it is noted that VSOR activity cannot attain RVD in acidic conditions, because depolarization caused by TRPM7/ASIC activation drives inflow of Cl^–^ and other small anionic osmolytes, but not volume-regulatory outflow of these anions, via VSOR, thereby exaggerating and persisting cell swelling (Figure 2A). To prevent generation of persistent cell swelling under acidic conditions, inhibition of either a single acid-sensitive anionic transmembrane pathway, ASOR, or two acid-sensitive cationic pathways, TRPM7 and ASIC, is necessary and sufficient because of the electroneutrality principle.

### Roles of ASOR/PAC in Acidotoxic Cell Death

Since TRPM7 is conductive to protons (Numata and Okada, 2008), sizable H^+^ inflow is induced by sustained extracellular acidosis and thereafter stimulates the Na^+^-H^+^ exchanger (NHE), thereby inducing additional Na^+^ accumulation within the cell (Figure 2B). Because the extracellular Na^+^ concentration is ten times higher than the intracellular one under physiological conditions, the electroneutral operation of Na^+^-H^+^ exchanger with the stoichiometry of 1:1 produces net Na^+^ influx and efflux, when the extracellular H^+^ concentration is higher and lower, respectively, than ten times of the intracellular one. When extracellular pH drops to 6 under acidosis, for example, net Na^+^ influx should be driven by H^+^ efflux even under mild intracellular acidification at pH below 7 via the Na^+^-H^+^ exchanger. NHE may also be activated by protons liberated from ATP hydrolysis, the rate of which inevitably overwhelms that of ATP synthesis under ischemic/hypoxic conditions. Further intracellular Na^+^ accumulation is caused by the operation of Na^+^-Ca^2+^ exchanger (NCX) upon the intracellular Ca^2+^ rise. Under physiological conditions with extracellular Ca^2+^ of 1 mM and the membrane potential of −62 mV at 37°C, for example, intracellular Ca^2+^ at the free concentration of over 100 nM should drive net Na^+^ influx via the electrogenic Na^+^-Ca^2+^ exchanger operating with the 3:1 stoichiometry. Such an intracellular Ca^2+^ rise may be caused by Ca^2+^ inflow due to acidosis-induced activation of Ca^2+^-permeable TRPM7 (Nadler et al., 2001; Monteilh-Zoller et al., 2003) and also of Ca^2+^-permeable ASIC (Waldmann et al., 1997; Chu et al., 2002; Xiong et al., 2004; Yermolaieva et al., 2004; Figure 2B). NCX may also be stimulated by Ca^2+^ entry via voltage-gated Ca^2+^ channels activated by depolarization caused by activation of ASOR, TRPM7, and ASIC in excitable cells. When extracellular acidosis persisted, the intracellular compartment may also become more acidic. For example, exposure to acidic bath solution with pH 6.0 for 30–80 min, the intracellular pH value was found to go down to 6.4–6.6 (Gerweck et al., 1999; Kittl et al., 2019). Proton permeability of TRPM7 (Numata and Okada, 2008) may partly contribute to the induction of intracellular acidification. Such intracellular acidosis is known to inhibit VSOR activity (Gérard et al., 1998; Sabirov et al., 2000), thereby completely disrupting the VSOR-mediated RVD mechanism (Figure 2B).

Tissue injury is often coupled to an acidic extracellular environment under pathological conditions and thus involves acid-induced cell death. Such acidotoxic cell death was actually observed in neurons and glial cells in the brain (Goldman et al., 1989; Siesjö et al., 1996; Ding et al., 2000) and epithelial and microvascular cells (Argenzio and Eisemann, 1996; Siesjö et al., 1996). The nature of acidotoxic cell death is mostly necrosis which is preceded by prolonged cell swelling, called NVI. Acidosis is also shown to induce necroptosis in neurons via ASIC1a independent of its ionic conduction (Wang et al., 2015b), and necroptotic cell death is also known to be coupled to NVI (Simard et al., 2012; Chen et al., 2016). The acidotoxic NVI event is initiated by parallel activation of anionic ASOR and cationic TRPM7/ASIC channels and becomes persisted by acid-induced dysfunction of volume-regulatory anionic VSOR channels (Figure 2B). Three different *in vitro* approaches have evidenced that acidotoxic cell death is triggered by persistent activation of ASOR: that is, first, prominent protection from necrosis was induced by pharmacological blockage of ASOR activation in human epithelial cells (Wang et al., 2007; Ullrich et al., 2019) and in mouse cortical neurons (Sato-Numata et al., 2014); second, that was attained by low temperature-induced inhibition of ASOR activity in mouse cortical neurons (Sato-Numata et al., 2014); and third, that was also attained after molecular downregulation of the ASOR core component TMEM206 by their knocking out in human epithelial cells (Ullrich et al., 2019; Yang et al., 2019) and by their gene silencing in rat cortical neurons and insulinoma cells (Osei-Owusu et al., 2020). *In vivo* application of DIDS, which is a known blocker not only for VSOR but also for ASOR, was found to attenuate ischemia-reperfusion-induced cell injury in hippocampal CA1 neurons (Inoue and Okada, 2007) and in myocardial cells (Wang et al., 2015a). However, there has been no *in vivo* study by pharmacological intervention specifically targeting ASOR. To prove that ASOR is a potential therapeutic target for many diseases involving acidotoxic necrosis, such *in vivo* pharmacological studies are warranted. It must be stressed that generation of acidotoxic necrosis might be experimentally or clinically prevented more feasibly by blocking a single Cl^–^ entry pathway, ASOR, than by simultaneously blocking four different Na^+^ entry pathways, TRPM7, ASIC, NHE, and NCX (Figure 2B).

### Roles of ASOR/PAC in Ischemic Lactacidotoxic Cell Death

Under physiological conditions, lactate can be rapidly metabolized by gluconeogenesis and by means of oxidation. However, as described in the preceding Part 1 article (Okada et al., 2020), ischemia/hypoxia results in not only proton accumulation but also lactate accumulation due to its production via enhanced anaerobic glycolysis-fermentation reactions (Siesjö, 1988; Marmarou, 1992). Anionic lactate ambiently accumulated under ischemic or hypoxic conditions is to be taken up with H^+^ via the monocarboxylate transporter (MCT) (Halestrap and Price, 1999), thereby causing intracellular lactate accumulation. Cell swelling produced by Na^+^ and Cl^–^ uptake mediated by acid-sensitive channels may be augmented by acidosis coupled to lactate accumulation, called lactacidosis (Figure 2C). Under lactacidosis, cell swelling was furthermore shown to be persisted in association with impaired VSOR activity both in neuronal (Mori et al., 2002) and in glial (Nabekura et al., 2003) cells. The MCT-mediated proton influx first stimulates NHE, thereby enhancing the NVI induction due to additional Na^+^ accumulation together with lactate accumulation within the cells, and it second exaggerates intracellular acidosis, thereby inhibiting VSOR activity and then inducing the RVD dysfunction. ATP depletion caused by ischemia/hypoxia also results in inhibition of VSOR activity by itself and by free Mg^2+^ liberation from Mg-bound ATP, because VSOR activity requires intracellular ATP in a manner independent of its hydrolysis (Jackson et al., 1994; Oiki et al., 1994) and is inhibited by cytosolic free Mg^2+^ rise (Oiki et al., 1994), as both were quantitatively shown in the previous review article (see **Figure 3** in Okada et al., 2019). Impaired VSOR activity such caused by ischemic lactacidosis induces prolonged cell swelling, NVI, which finally results in necrotic cell death called lactacidotoxic necrosis, as depicted in Figure 2C. In order to prevent lactacidotoxic necrosis, either simultaneous inhibition of two different anionic entry pathways, ASOR and MCT, or that of four different Na^+^ entry pathways, TRPM7, ASIC, NHE, and NCX, is required.

Since the highest molecular expression of ASOR/PAC core molecule, TMEM206, in human tissues was found in the cerebral cortex (Yang et al., 2019), ASOR/PAC activity can be deemed to be essentially involved in ischemic brain injury *in vivo*. In fact, ischemic brain infarct induced by permanent middle cerebral artery occlusion (pMCAO) was found to become significantly diminished, but not completely eliminated, in TMEM206 knockout mice by Yang et al. (2019).

It is known that hypothermia is a most effective therapeutic means for protecting the ischemic and traumatic brain injury (Gunn and Thoresen, 2006; Liu and Yenari, 2007; Yenari and Han, 2012). Involvements of TRPM7/ASIC cation channels in hypothermia are unlikely, because temperature sensitivity of TRPM7 has never been shown (Huang et al., 2006; Wang and Siemens, 2015) and the ASIC current amplitude is not temperature-dependent (Neelands et al., 2010). In contrast, ASOR anion channels are known to be highly sensitive to temperature in epithelial (Sato-Numata et al., 2013) and neuronal (Sato-Numata et al., 2014) cells. Thus, the main effector of hypothermia therapy for acidotoxic neuronal cell death and for ischemic/hypoxic brain injury is likely to be the neuronal ASOR/PAC anion channel (Figures 2B,C). Actually, not only acidotoxic cell swelling (NVI) but also necrotic cell death was found to be largely subsided by lowering temperature to 25°C in cortical neurons (Sato-Numata et al., 2014).

## Conclusion and Perspective

In conclusion, ASOR/PAC is ubiquitously expressed, is formed by a homo-trimer of TMEM206, and exhibits phenotypic properties. In spite of the recent pronounced progress of ASOR/PAC/TMEM206 studies, its open-state structure and detailed molecular mechanisms for its activation, including intracellular signaling, remain to be elucidated.

Under physiological conditions, ASOR/PAC anion channels serve as a proton sensor together with TRPM7 and ASIC cation channels in mammalian cells. Under pathological conditions coupled to persistent acidosis, such as ischemia/hypoxia situations, ASOR/PAC activity is essentially involved in sequential induction of persistent cell swelling, acidotoxic necrosis and lactacidotoxic necrosis. Since persistent cell swelling is known to be an early prerequisite process for induction of necrotic cell death, this is called NVI and to be an effective therapeutic target. It must be stressed that under acidotoxic conditions, ASOR/PAC represents a single key player as an acid-sensitive transmembrane anion entry pathway for NVI induction. In contrast, four types of channels/transporters, TRPM7, ASIC, NHE, and NCX, serve as the pathways for NVI-inducing transmembrane Na^+^ entry under acidotoxic conditions. Thus, the future development of ASOR/PAC blocking agents, that are specific, less toxic and blood-brain barrier-permeable, is awaited for clinical use. Also, it must be noted that keen temperature sensitivity of ASOR/PAC may provide a most promising target for hypothermic therapy of ischemic/hypoxic brain injury.

## Author Contributions

YO conceived of the project. YO, KS-N, and RS wrote the manuscript with input from all authors. RS and TN prepared figures. KS-N prepared references. All authors contributed to the article and approved the submitted version.

## Conflict of Interest

The authors declare that the research was conducted in the absence of any commercial or financial relationships that could be construed as a potential conflict of interest.
